# Supplementary material for: A taxon-specific measurement of disruption in a multi-modal study of microbiomes and metabolomes reveals system-wide dysbiosis preceding HIV-1 infection
Source: Nat Commun. 2025 Nov 20;16:10204. doi: 10.1038/s41467-025-64822-z (PMC12635353; doi:10.1038/s41467-025-64822-z)
Supplement: Supplementary file 1 — Supplementary Information [file 41467_2025_64822_MOESM1_ESM.pdf]

Supplementary Information

A taxon-specific measurement of disruption in a multi-modal study of microbiomes and metabolomes reveals system-wide dysbiosis preceding HIV-1 infection

Fouladi F, Chen Y, Bera S, Jarmusch AK, Van Tyne D, Palella FJ, Margolick JB, Chew KW, Sun J, Martinson J, Rinaldo CR, Peddada SD*

Methods

Metabolomics

Following steps were performed by Creative Proteomics, Inc. NY, USA, www.creative-proteomics.com using the MACS plasma, oral, and stool samples provided Rinaldo Lab*:*

Sample preparation: Following thawing samples on ice, 100 mg stool sample was transferred in a tube and added with 800 µL 80% methanol. All samples were vortexed for 30 s, ground for 90 s at 65 Hz, and then sonicated for 30 min at 4°C. Then each sample was kept at -20°C for 1 h, vortexed for 30 s and kept at 4°C for 30 min. After that, samples were centrifuged at 12,000 rpm and 4°C for 15 min. All the supernatant was transferred to a new tube, keep at -20°C for 1 h, centrifuged at 12,000 rpm and 4°C for 15 min. Finally, 200 µL of supernatant was transferred into vial and 5 µL of 0.14 mg/mL DL-o-Chlorophenylalanine as internal standard was filtered through a 0.22 µm filter LC-MS analysis. A similar sample preparation procedure was used for oral and plasma samples, except for the first step where 300 µL 80% methanol was added to 100 µL thawed samples.

Liquid Chromatography-Mass Spectrometry: Metabolomic analysis was performed using ultra-high performance liquid chromatography (Vanquish Flex UPLC) connected to an Orbitrap Q Exactive mass spectrometer equipped with electrospray ionization (Thermo Fisher Scientific, USA). For UPLC separation, 0.05% formic acid in water was prepared as mobile phase A and acetonitrile was prepared as mobile phase B. The solution was transferred to a liquid chromatography (LC) vial and kept in the auto-sampler at 4°C until analysis. 5 µL of solution was loaded into the UPLC and separated on a T3 reversed phase column (ACQUITY UPLC HSS T3, 2.1*100 mm, 1.8 µM). Chromatographic separation was performed at a flow rate of 0.3 mL/min and 40°C. The elution gradient is as follows, 0-1.0 min, 5% B; 1.0-12.5 min, 5%-95% B; 12.5-13.5 min, 95% B; 13.5-13.6 min, 95%-5% B; 13.6-16 min, 5% B.

Metabolomic analyses were performed in both positive (ESI+) and negative (ESI-) electrospray ionization modes. The electrospray ionization positive mode was operated using following conditions: heater temperature of 300°C, sheath gas flow rate of 45 arb, aux gas flow rate of 15 arb, sweep gas flow rate of 1 arb, spray voltage of 3.0 KV, capillary temperature of 350°C and S-Lens RF level set constant at 30%. However, in ESI negative mode, spray voltage and S-Lens RF level were set at 3.2 KV and 60%, while other conditions remained same as the ESI positive mode. The full scan was performed 70-1050 m/z at the resolution of 70,000 fwhm, automatic gain control (AGC) target of 3×106, maximum injection time of 100 ms and spectrum data type of centroid. Significant features were identified using data-dependent MS2. Settings for dd-MS2 data acquisition was as follows, resolution of 17,500 fwhm, AGC target of 1×105, maximum injection time of 50 ms, isolation window of 1.7 m/z, loop count of 10, and normalized collision energy (NCE) was set at 15, 30 and 45 eV, spectrum data type of centroid.

Analytical Processes: The Compound Discoverer 3.1 (CD3.1, Thermo Fisher) software was used to process raw data generated by the UPLC-MS via peak alignment, peak picking, and identification of each metabolite. After analysis, these data were matched with the mzCloud, mzVault databases to obtain accurate identification results. The data was further processed by Progenesis QI to provide strong support for identifying metabolites accurately.

Data Imputation: Fill Gaps node was used to find chromatographic peaks that were detected by the Detect Compounds node in one of the input files but were missing from other input files in the file set. The following steps were followed for gap filling*:*

1. Detection limit is calculated for each missing ion as an area of a simulated Gaussian peak that starts and ends at a zero-intensity baseline. To calculate the area of the Gaussian peak, the node uses the expected peak width and the maximum spectrum noise in the expected retention time range multiplied by the S/N threshold.
2. Missing ions are searched with the expected m/z × RT dimensions against all detected ions while ignoring the assigned adduct type. If it finds a match (ion with the expected m/z × RT dimensions), it uses the ion's area to fill the gap and displays Filled by Matching Ion for the Fill Status.
3. If the node does not find a matching ion, it attempts to detect the peak at a lower intensity threshold using the Parameterless Peak Detection (PPD) algorithm. If it detects a chromatographic peak at a lower threshold, it uses the integrated peak area to fill the gap and displays Re-detected Peak for the Fill Status.
4. If the node does not find a chromatographic peak by using a lower intensity threshold, it fits a Gaussian peak to the XIC trace for the expected m/z range and displays Filled by Simulated Peak for the Fill Status.
5. If the filled area is still zero or lower than the detection limit, the node uses the detection limit value to fill the gap and displays Filled by Spectrum Noise for the Fill Status.
6. If the node cannot fill the gap, it displays Area Could Not Be Filled for the Fill Status.

Short chain fatty acids (SCFA)

Each sample was diluted 5 or 10-fold with wash solution and vortexed well. Samples were diluted in water containing labelled internal standards for each chain length (C2-C6). The free short chain fatty acids were derivatized using methyl chloroformate in 1-propanol yielding propyl esters before subsequent liquid-liquid extraction into hexane and analysis on a SLB-5ms (30 × 0.25 mm × 1.0 µm) column and detection using GC-EI-MS in SIM-mode. The analytes were quantified using 6-point calibration curves. SCFAs were measured by *Creative Proteomics*, Inc. (NY, USA).

DISCO – Validation study

In addition to the validation of DISCO using external datasets, we also evaluated its performance by generating null data and non-null data from the gut species abundance table using the MACS cohort as follows:

Null data (A): We combined the Pre-HIV group (n_1 = 86) and Non-HIV group (n_2 = 149) into a single population and then randomly resampled the data with replacement into two groups of sizes n_1 = 86 and n_2 = 149. We then computed the DISCO score for each taxon between two randomly created groups. We repeated this process 1000 times. Since the Cauchy random variable has no mean, we computed the median DISCO score for each taxon over the 1000 times resampling.

Non-null – Real data (B): We drew a random sample with replacement of size 86 from the Pre-HIV group (n_1 = 86) and a random sample with replacement of size 149 from the Non-HIV group (n_2 = 149). As in (A), we then computed the DISCO score for each taxon between two randomly created groups. We repeated this process 1000 times. Thus, we bootstrapped samples within the real data. Again, since the Cauchy random variable has no mean, we computed the median DISCO score for each taxon over the 1000 times resampling.

The median DISCO scores from null and non-null data are shown in Supplementary Figure 11. Since data in (A) are created by mixing the samples from the two groups, the resulting DISCO score for each taxon can be viewed as the score under the Null, whereas since the DISCO score for data in (B) are based on two different phenotypes, it may be regarded as score under the non-null. As expected, the distribution of median DISCO scores of taxa derived from real data is stochastically larger than that derived from null data. The top DISCO scores from the real data belonged to *Mogibacterium kristiansenii*, *Prevotella sp. P5_64*, *Bacteroides thetaiotaomicron*, *Blautia faecis*, *Holdemanella biformis*, *Phascolarctobacterium succinatutens*, and *Oscillibacter sp. ER4*. All these species have a significant DISCO score in Figure 4.

Results

As in the main text, LFCs are provided within parenthesis and unless stated otherwise, the significance of a finding is based on $p < 0.01$ and $q < 0.1$.

Oral Microbiome dysbiosis

Overall, we observed fewer differences in the oral microbiome and metabolome of Pre-HIV (relative to Non-HIV) compared to their gut microbiome and metabolome. For example, we observed no oral microbial species differentially abundant between Pre-HIV and Non-HIV at $q < 0.1$ (Supplementary Figure 4A, Supplementary Data 6A). Nonetheless at $p < 0.01$, we did find lower abundance of oral commensal bacteria *Tannerella serpentiformis* (-0.82, $q = 0.15$) [1] and *Lautropia mirabilis* (-1.16, $q = 0.20$) [2] among Pre-HIV and an increase in proinflammatory species, including *Porphyromonas endodontalis* (1.16, $q = 0.12$) [3] and *Candidatus Nanosynbacter sp. TM7 075* (0.79, $q = 0.12$) [4,5]. Higher abundances of the latter two species are associated with periodontal disease, a disease commonly seen among PWH.

We also did not find many different gene functions corresponding to the oral microbial gene functions at $q < 0.1$. However, using raw p-values ($p < 0.01$), we found 13 functional terms reduced, and 21 functional terms enriched, in Pre-HIV (Supplementary Figure 4B, Supplementary Data 6B). Specifically, we found lower levels of glutamate dehydrogenase (NAD+) activity (-0.64, $q = 0.24$) and glutamate catabolic process to 2-oxoglutarate (-0.64, $q = 0.24$) which are involved in the deamination of glutamate and play an important role in carbon and nitrogen metabolism. Lower trehalose transmembrane transporter activity (-0.40) among Pre-HIV was also observed, which has several critical functions including protection against oxidative damages by reactive oxygen species [6,7]. On the other hand, there was a higher level of the formate metabolic process (0.37), which could be involved in immune response and inflammation [8,9].

To evaluate whether these differences in the oral microbiome between Pre-HIV and Non-HIV could be associated with sexual activity, we performed trend analysis of oral species and their gene functions over four sexual activity groups defined as the number of partners with whom a participant had receptive anal intercourse (Supplementary Data 7). Interestingly, although *Candidatus Nanosynbacter sp. TM7 075* tended to be associated with Pre-HIV, its abundance tended to decrease with a higher number of receptive anal sexual partners. In contrast, *Aggregatibacter sp. oral taxon 458,* tended to be associated with both Pre-HIV and number of receptive anal sexual partners (Supplementary Figure 5A, Supplementary Data 7A). We also found 42 gene functions associated with sexual activity ($p < 0.01$, 19 gene functions with $q < 0.1$, Supplementary Figure 5B, Supplementary Data 7B). Most of the gene functions associated with Pre-HIV seemed to be also associated with sexual activity except for formate metabolic process and a few gene functions related to sugar metabolism (such as rhamnose metabolic process, fructose 2,6−bisphosphate metabolic process, glucose−1−phosphate cytidylyltransferase activity) (Supplementary Figure 6B).

We did not observe any significant differences in the oral metabolome between Pre-HIV and Non-HIV or any significant patterns over sexual the activity groups at $q < 0.1$. Oral acetic acid and propionic acid tended to be higher in Pre-HIV compared to None-HIV ($p < 0.05$) (Supplementary Figure 4C).

As with gut microbial species, we performed differential correlation analyses of oral microbial species with other oral microbial species (Supplementary Data 8A), with oral microbial functional terms (Supplementary Data 8B), with the oral metabolome (Supplementary Data 8C and 8D) and with the plasma metabolome (Supplementary Data 8E and 8F). Several interactions were significantly different between Pre-HIV and Non-HIV across all modalities ($p < 0.01$, $q < 0.1$, differences in absolute value of correlations > 0.30). To determine whether sexual activity contributed to these differential correlations, we performed trend analysis of pairs of features differentially correlated between Pre-HIV and Non-HIV over the four sexual activity groups. In this section, we summarize some of the important findings.

Differences in correlations among oral species between Pre-HIV and Non-HIV did not survive multiple testing at $q < 0.1$, however, there were a few differences with $p < 0.01$ and $q < 0.2$ which could be biologically relevant. Notably, oral species within the genus *Rothia* (*R. mucilaginosa*, *R. dentocariosa*, *R. sp. HMSC062F03*, *R. sp. HMSC065C12*, and R. *sp. HMSC072E10*) were positively correlated with *Streptococcus spp.* in Preh-HIV ($p < 0.001$, $q = 0.1$) (Supplementary Data 8A). *Rothia* and *Streptococcus* are part of the normal flora of oral cavity, however, some species within these genera are opportunistic pathogens and are associated with dental caries and periodontal disease [10]. Especially, *Rothia mucilaginosa* is associated with bacteremia in immunocompromised hosts [11]. In our study, *Rothia dentocariosa*, *Streptococcus sp. HPH0090,* and *Streptococcus sp. SK140* were positively associated with oral and plasma cotinine (Supplementary Data 9C and 8E, $q < 0.1$). Previously, it was shown that tobacco users had higher abundances of *Rothia* and *Streptococcus* in their saliva compared to tobacco non-users [12,13]. These results indicate external factors, such as smoking, could contribute to oral dysbiosis in Pre-HIV.

We observed *Candidatus Nanosynbacter sp. TM7_075,* which tended to be lower in Non-HIV, had a positive relationship with *Schaalia odontolytica* (formerly known as *Actinomyces odontolyticus*, $p = 0.001$ $q = 0.11$*)* and *Actinomyces graevenitzii* ($p = 0.003$, $q = 0.18$) in the Non-HIV group but not in the Pre-HIV group (Supplementary Data 8A). *Candidatus Nanosynbacter Unclassified TM7 075* belongs to the phylum *Saccharibacteria*, formerly known as *TM7*, which are obligate epibionts living on the surface of oral bacteria [14,15], such as *Actinomyces odontolyticus*. They are associated with dysbiotic oral microbiome and oral diseases, such as periodontitis [4,5] and gingivitis [16,17], as well as other inflammatory diseases, such as vaginosis, IBD, and lung diseases (Table S1 in [14]). Using a ligature-induced periodontitis mouse model, Chipashvili et al. [14] showed that *TM7* reduces the pathogenicity of their host bacteria that they live off to prevent stimulation of the immune system and thereby to ensure their own survival. Thus, it is possible that any perturbation in the healthy interaction between *TM7* with their bacterial host could turn their bacterial host more virulent and pathogenic, resulting in increased inflammatory responses and disrupted oral microbial homeostasis. Inflammation could deplete the health-associated or commensal bacteria that cannot thrive in such an inflammatory condition [18].

A striking finding is that *Haemophilus parainfluenzae* was negatively correlated with 76 unique functional terms in Non-HIV, but these relationships were attenuated in Pre-HIV ($q < 0.1$). Since *Haemophilus parainfluenzae* is an opportunistic pathogen, its negative correlations with oral microbiome in Non-HIV may indicate that commensal bacteria could inhibit overgrowth of this pathogenic bacterium, while this relationship might be missing in the dysbiotic oral microbiome in Pre-HIV (Supplementary Figure 7A, Supplementary Data 9A). Most of these correlations showed a decreasing trend over the sexual activity groups (Supplementary Figure 8A), suggesting that disruption in relationships between *Haemophilus parainfluenzae* and oral microbiome could be related to sexual activity.

We observed that several species of *Streptococcus*, including *S. infantis*, *S. sp. Marseille_Q4154, S. sp. oral_taxon_431, S. sp. Marseille_Q6470, S. sp. Marseille_Q0941, and S. sp. F0442,* were all negatively correlated with 1-hexadecanoyl-sn-glycero-3-phosphocholine (LPC 16:0) in Pre-HIV (Supplementary Figure

7B, Supplementary Data 9B). These relationships did not change by the sexual activity groups (Supplementary Figure 8B). LPC 16:0 is lysophosphatidylcholine with a palmitic acid (16:0) chain and has proinflammatory properties [19]. This could indicate that an increase in pro-inflammatory oral metabolites reduces the abundance of several commensal *Streptococcus* species.

Our results provide evidence that the interactions between the gut and oral microbiomes are significantly different between Pre-HIV and Non-HIV. We found 55 correlations between oral and gut species to be different between Pre-HIV and Non-HIV (Supplementary Data 9F). These differential correlations included mostly gut species within the genera *Eubacterium* (5 correlations), *Bifidobacterium* (5 correlations), and *Roseburia* (4 correlations), and oral species within the genera *Streptococcus* (13 correlations), *Porphyromonas* (6 correlations), and *Prevotell*a (6 correlations). Species belonging to these genera are shown in Supplementary Figure 7D. Except for reduced correlation between gut *Prevotella copri* and oral *Prevotella salivae* in Pre-HIV, no other differential correlations included gut and oral species from the same genus. On the other hand, there were several plasma metabolites interacting with gut and oral microbiomes differently among Pre-HIV and Non-HIV. Among them, 4-ethylphenylsulfate, a microbiome-derived metabolite in the gut associated with autism [20,21], was negatively correlated with oral *Prevotella spp.* and positively correlated with oral *Streptococcus sp. XMC* (Supplementary Figure 7C, Supplementary Data 9D) in Pre-HIV. These results suggest oral and gut microbiomes could indirectly interact with each other through plasma metabolites.

Application of DISCO to External datasets

The proposed DISCO score is illustrated using four external datasets. All data were normalized and analyzed using the methods described in the Methods section of this paper. For metagenomic datasets, analyses were performed at the species level, whereas for 16S datasets, analyses were performed at the lowest known taxonomic level. In the following, first we describe each dataset and then explain the findings.

Armstrong et al. 2018 [22]*:* The authors in this study characterized the differences in the gut microbiome composition between MSM (35 HIV-1 negative) and non-MSM (29 MSW HIV-1 negative and 41 women HIV-1 negative), as well as HIV-associated changes in MSM (90 HIV-1 positive) and non-MSM (19 women HIV-1 positive and 3 MSW HIV-1 positive) using 16S rRNA gene sequencing. Amplicon Sequence Variant (ASV) frequency tables retrieved from https:/qiita.ucsd.edu/, and ASVs were taxonomically classified using the SILVA138 database[23]. Since almost all the HIV-1 positive participants in this study were MSM, we computed DISCO scores only for the MSM group (32 HIV-1 negative versus 39 antiretroviral therapy (ART)-naïve HIV-1 positive and 50 ART-treated HIV-1 positive). Taxa that were prevalent in less than 30% of samples in each group were removed prior to downstream analyses. Data were also corrected for the potential confounding effect of age.

Fulcher et al. 2022 [24]*:* This study includes 27 individuals before and after HIV-1 infection from three longitudinal cohorts of MSM in the US (mSTUDY, The Healthy Young Men's Cohort Study (HYM), and RADAR) and one longitudinal cohort in Peru (Rectal STI study). The control group includes 28 participants without HIV-1 but with a similar behavioral risk, selected from the same cohort studies. The majority of participants were Hispanic/Latino and Black. The gut microbiome was characterized using shotgun metagenomic sequencing from longitudinal rectal swab samples. Metagenomic sequences from 27 controls, 27 pre-HIV samples, and 27 post-HIV samples were obtained from NCBI PRJNA836336 and processed using the JAMS pipeline as described for the MACS cohort in this paper. To remove the effect of location, residuals of taxa abundances were obtained from fitted linear models with country (USA and Peru) as an independent variable and taxa abundance as a dependent variable. Correlations among gut

species as well as between species and gene functions were compared between the control group and the pre-HIV group, as well as between the control group and the post-HIV group.

Garcia et al. 2024 [25]: This is a cross-sectional study of 129 individuals with different HIV-1 profiles (concordant, discordant, early treated, elite controller, late presenter, ART-naïve, viremic controller) and 27 HIV-1 negative controls recruited in Barcelona, Spain. The goal of the study was to characterize the relationship between the gut resistome and HIV-1 infection using shotgun metagenomics. To apply the DISCO score to this study, we obtained metagenomes from 100 MSM, including 77 HIV-1 positive and 23 HIV-1 negative individuals from NCBI PRJNA307231 and processed the sequences using the JAMS pipeline as described for the MACS cohort in this paper. Following data normalization, residuals of taxa abundances were obtained from fitted linear models with ethnicity and HIV-1 profile as independent variables to remove their potential confounding effects.

Rocafort et al. 2024 [26]: The aim of this study was to compare the microbial signature associated with HIV-1 infection between three different countries: the US (n = 233), Uganda (n = 170), and Botswana (n = 194). The fecal microbiome was characterized using 16S rRNA gene sequencing. Processed data and metadata were obtained from Supplementary Data 1, 2, and 3 from the original paper. We performed differential correlation analysis and computed DISCO scores using the MSM Boston cohort (32 HIV-1 negative and 86 HIV-1 positive), the non-MSM Boston cohort (85 HIV-1 negative and 30 HIV-1 positive), the Botswana cohort (80 HIV-1 negative and 114 HIV-1 positive), and the Uganda cohort (80 HIV-1 negative and 90 HIV-1 positive). For all the cohorts, taxa that were prevalent in less than 30% of samples in each group were removed prior to downstream analyses. Data were also corrected for the potential confounding effects of age, ethnicity, and gender (in the case of non-MSM).

Applying DISCO to the gut metagenomics data in Fulcher et al. 2022 [24], consisting of 27 MSM pre- and post-HIV and their matched controls, we found that several species, such as *Prevotella bivia*, *Prevotella ihumii*, *Prevotella sp. 885*, *Faecalibacterium prausnitzii*, and *Streptococcus mitis*, were disrupted in their correlations both prior to and after HIV-1 infection (Supplementary Figure 9A, Supplementary Data 11A-D). Some species, such as *Oscillibacter valericigenes*, were significantly disrupted only prior to HIV-1, whereas others, such as *Dorea formicigenerans*, were only disrupted after HIV-1, suggesting that the gut microbiome could undergo changes following HIV-1 infection. For a second illustration, we applied DISCO to the gut microbiome data in Garcia et al. 2024 [25], consisting of 77 MSMs with HIV-1 and 23 MSMs without HIV-1. DISCO identified *Prevotella spp*. and several species belonging to the *Proteobacteria* family including *Succinivibrio spp.,* and *Sutterella wadsworthensis* to be significantly disrupted in their correlations in participants with HIV-1 infection (Supplementary Figure 9B, Supplementary Data 11E-F). Rocafort et al. [26] conducted a multinational study of the gut microbiome of participants with HIV-1 living in USA (Boston), Uganda, and Botswana. The MSM Boston cohort included 32 HIV-1 negative and 86 HIV-1 positive participants, and the non-MSM Boston cohort included 85 HIV-1 negative and 30 HIV-1 positive participants. The Botswana cohort consisted of 80 HIV-1 negative and 114 HIV-1 positive participants, and the Uganda cohort included 80 HIV-1 negative and 90 HIV-1 positive participants. Rocafort et al. [26] reported that taxa associated with HIV-1 infection differed between MSM and non-MSM as well as across the three locations. Interestingly, we observed a signature of disrupted species unique to each cohort (Supplementary Figure 9C, Supplementary Data 11G-J). Botswana had the smallest number of significant disrupted species, an observation which is consistent with the DAA reported in [26]. In our analysis, *Prevotella copri* was significantly disrupted in both Uganda and MSM-Boston cohorts. This result supplements Rocafort et al.'s finding that *Prevotella copri* differentially abundant between HIV-1 negative and HIV-1 positive participants in both cohorts, but with different directions; it was higher in HIV-1 positive participants in the MSM-Boston cohort while higher in HIV-1 negative participants in Uganda. In [22],

Armstrong et al., studied the differences in the microbial compositions of HIV-1 positive MSM who were treated by anti-retroviral therapy (ART-treated) and who were not (ART-naïve). Applying DISCO to these data, we found that the ART status could affect the gut microbiome as some genera or species were found to be disrupted in their correlations in individuals treated with ART, while others were disrupted in the ART-naïve group (Supplementary Figure 9D, Supplementary Data 11K-L). Interestingly, *Bacteroides* was significantly disrupted in their correlations in individuals with HIV-1 regardless of ART status. Finally, we compared the number of disrupted species within each genus across all the cohorts, including MACS cohort reported in this paper. We found that *Prevotella spp.* had the highest number of species that were disrupted in their correlations across most cohorts, followed by *Blautia*, *Dorea*, *Oscillibacter*, and *Bacteroides* (Supplementary Figure 10).

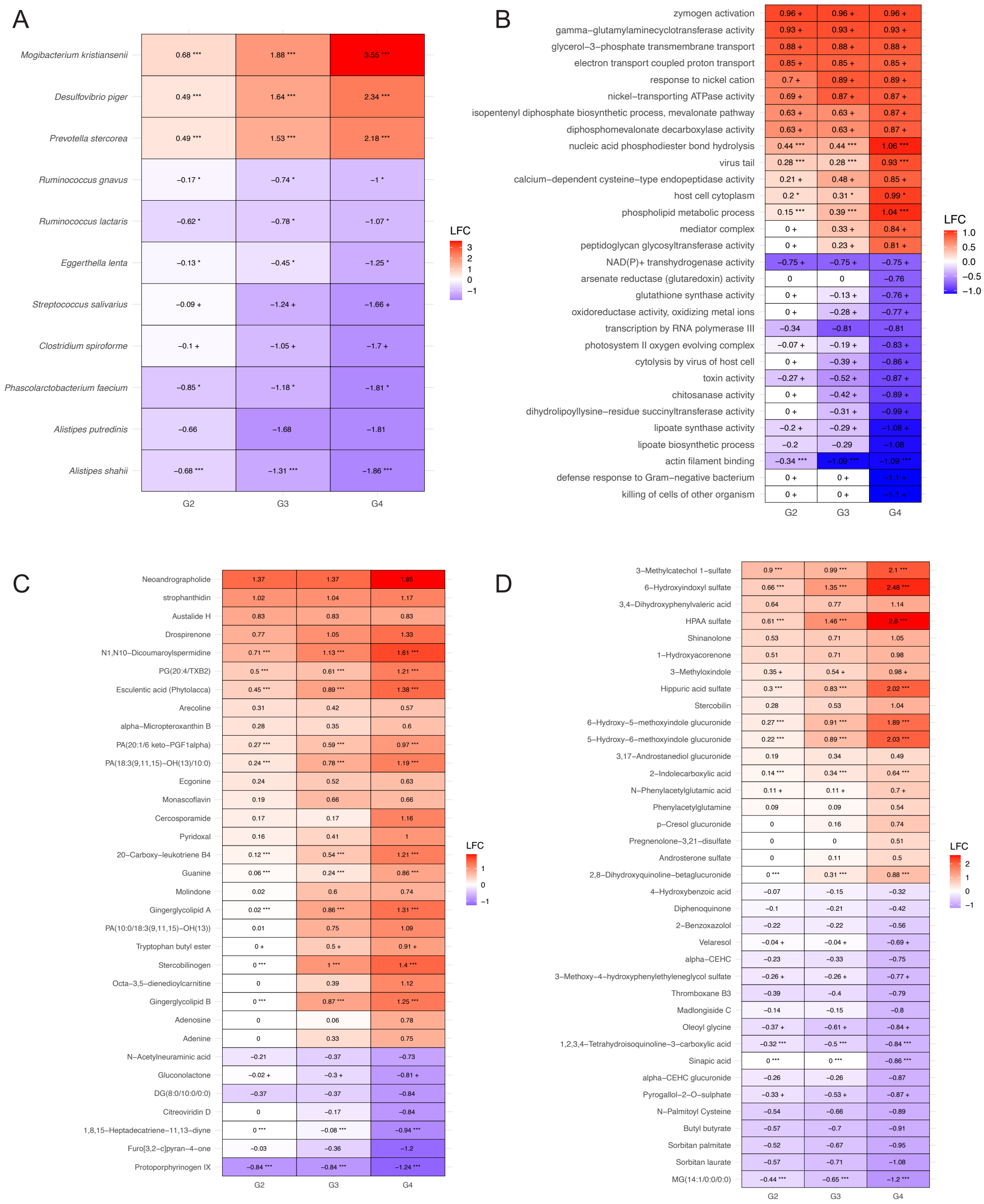

Supplementary Figure 1 (Trend analysis of gut microbiome and gut and plasma metabolites over sexual activity groups). Trend analysis over four sexual activity groups defined as the number of partners with whom a participant had receptive anal intercourse: G_1, G_2, G_3, G_4 corresponds to groups with 0, 1, 2-5, and 6 or more receptive anal intercourse partners, respectively. Log-fold changes of absolute abundances of features in G_2, G_3, and G_4 relative to G_1 are presented for statistically significant differentially abundant features ($p < 0.01$) with + $q < 0.1$, * $q < 0.05$, ** $q < 0.01$, *** $q < 0.001$. The red and blue tiles indicate that the abundances of features have an increasing and decreasing trend over the sexual activity groups, respectively. These results are obtained by applying ANCOM-BC2. **A.** Gut bacterial species. **B.** Gut microbial Gene Ontology (GO) terms, only the top 30 significant GO terms are shown in this figure. **C.** Gut metabolites. **D.** Plasma metabolites. The sample sizes vary by data modality as described in Supplementary Data 12.

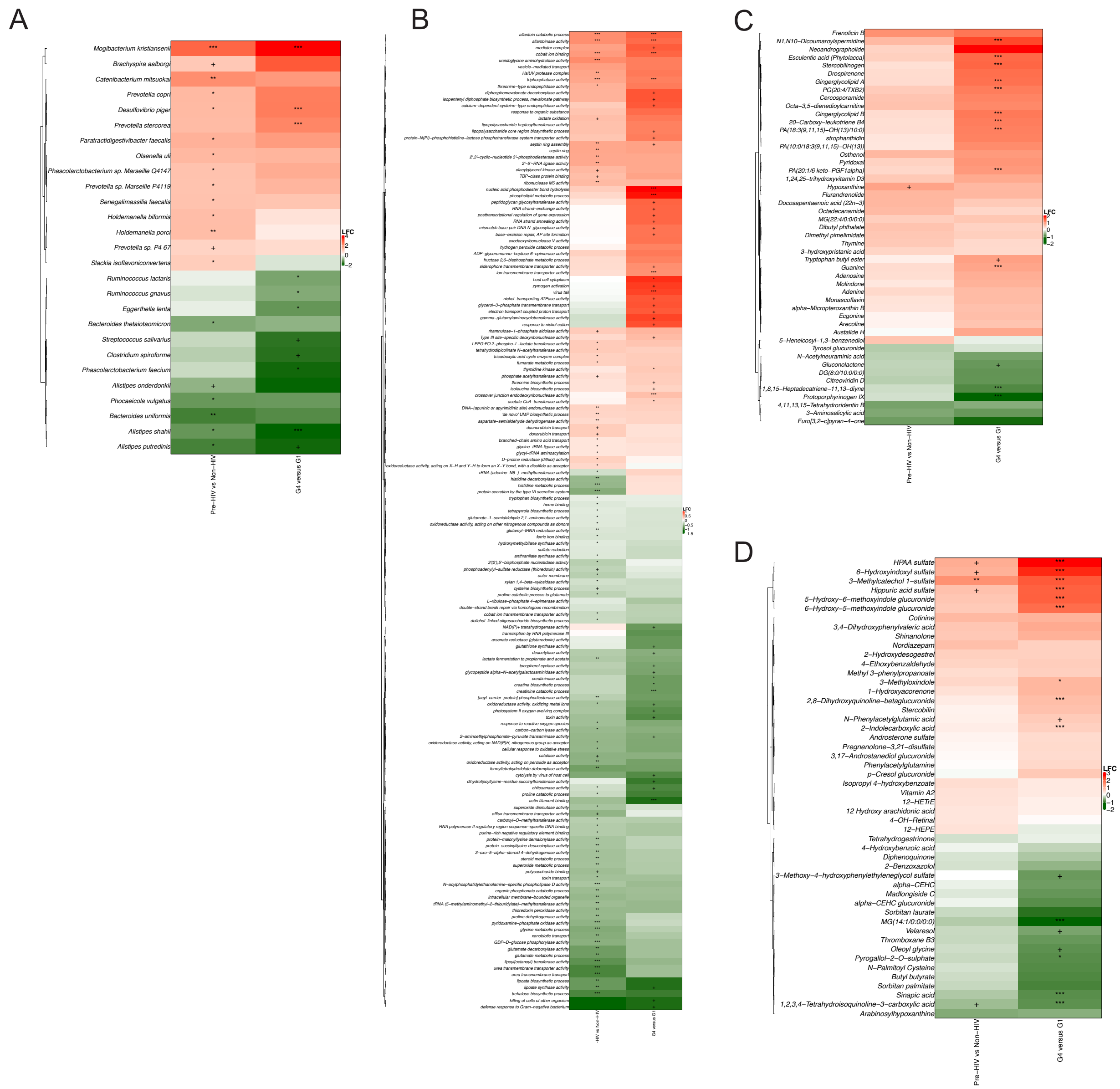

Supplementary Figure 2 (Differential abundance analysis of gut microbiome and gut and plasma metabolites between Pre-HIV and Non-HIV versus trend analysis over sexual activity groups). Heatmaps show significant features at $p < 0.01$ from differential abundance analysis (DAA) between Pre-HIV and non-HIV and trend analysis over four sexual activity groups. + $q < 0.1$, * $q < 0.05$, ** $q < 0.01$, *** $q < 0.001$. For DAA, log-fold changes of absolute abundances of features in Pre-HIV relative Non-HIV are presented. For trend analysis, although the p-values are derived using all four groups G_1, G_2, G_3, G_4, for graphical representation, the log-fold changes of absolute abundances of features in the extreme groups G_4 relative to G_1 are presented. These results are obtained by applying ANCOM-BC2. **A.** Gut bacterial species. **B.** Gut microbial Gene Ontology (GO) terms. **C.** Gut metabolites. **D.** Plasma metabolites. The sample sizes vary by data modality as described in Supplementary Data 12.

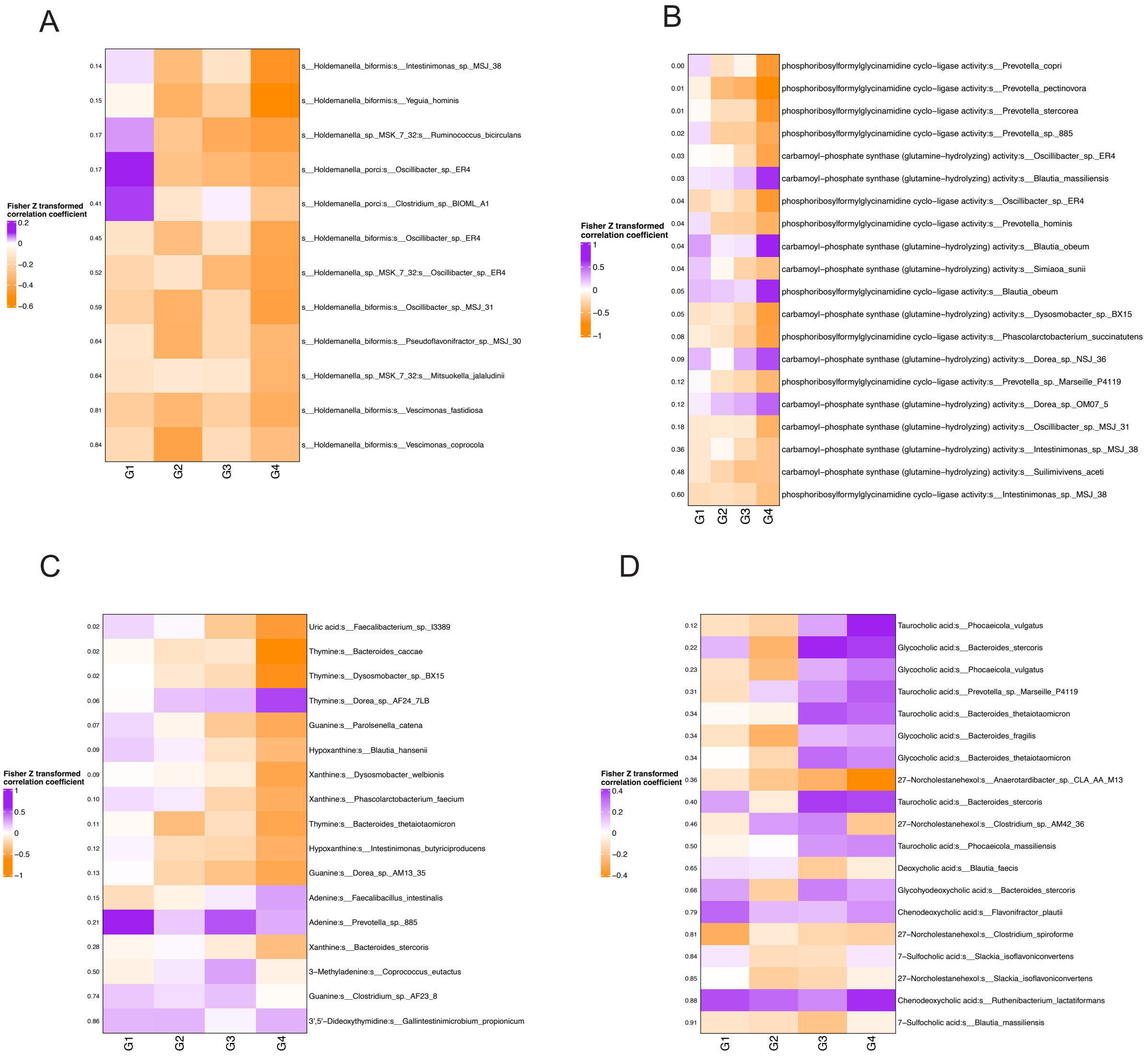

Supplementary Figure 3 (Heatmaps of correlations among features related to gut microbiome across sexual activity groups). Heatmaps show Spearman correlation coefficients of pairs of features that were differentially correlated between Pre-HIV and Non-HIV as shown in Figure 3 for each sexual activity group. Benjamini-Hochberg adjusted p-values from the trend analysis (PAVA) are shown. **A.** Correlations between *Holdemanella* spp. and other gut species. **B.** Correlations between Gene Ontology (GO) terms related to microbial purine and pyrimidine biosynthesis pathways and gut species. **C.** Correlations between gut purine and pyrimidines derivatives and gut species. **D.** Correlations between plasma bile acids and gut species. The sample sizes vary by data modality as described in Supplementary Data 12.

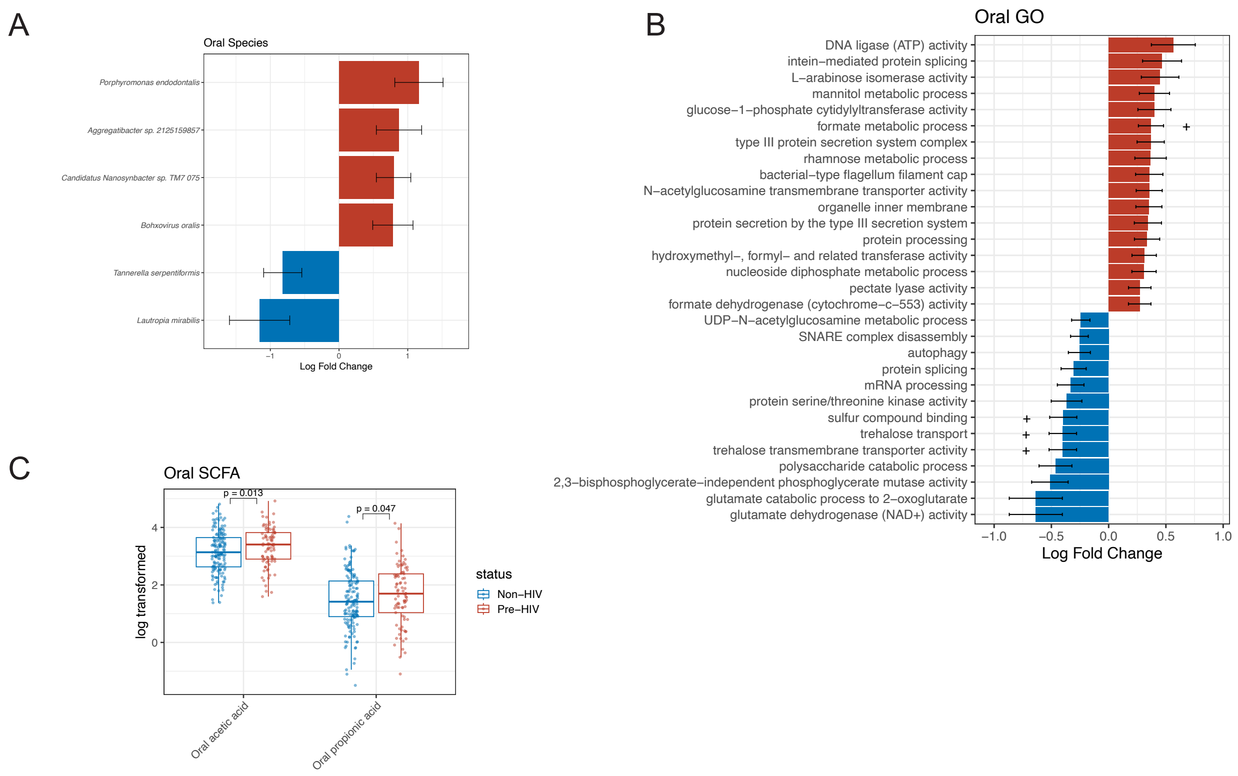

Supplementary Figure 4 (Differential abundance analysis (DAA) of oral microbiome and oral short chain fatty acids between Pre-HIV and Non-HIV). A-B. Log-fold changes of absolute abundances are presented for statistically significant differentially abundant features ($p < 0.01$). The red bars to the right of 0 axis correspond to higher abundance in Pre-HIV and blue bars to the left of 0 axis correspond to decrease in abundance in Pre-HIV. Features that are also significantly different after multiple testing correction using BH procedure are represented by + $q < 0.1$, * $q < 0.05$, ** $q < 0.01$, *** $q < 0.001$. These results are obtained by applying ANCOM-BC2. **A.** DAA of bacterial species. **B.** DAA of gut microbial Gene Ontology (GO) terms. **C.** DAA of oral short chain fatty acids. The center line of the boxplot represents the median, the box shows the interquartile range (IQR: Q1–Q3), and the whiskers extend to the most extreme values within 1.5 × IQR from the box. Data points beyond the whiskers are plotted as outliers. The significance of gut and plasma results is based on linear regression analysis. The sample sizes vary by data modality as described in Supplementary Data 12.

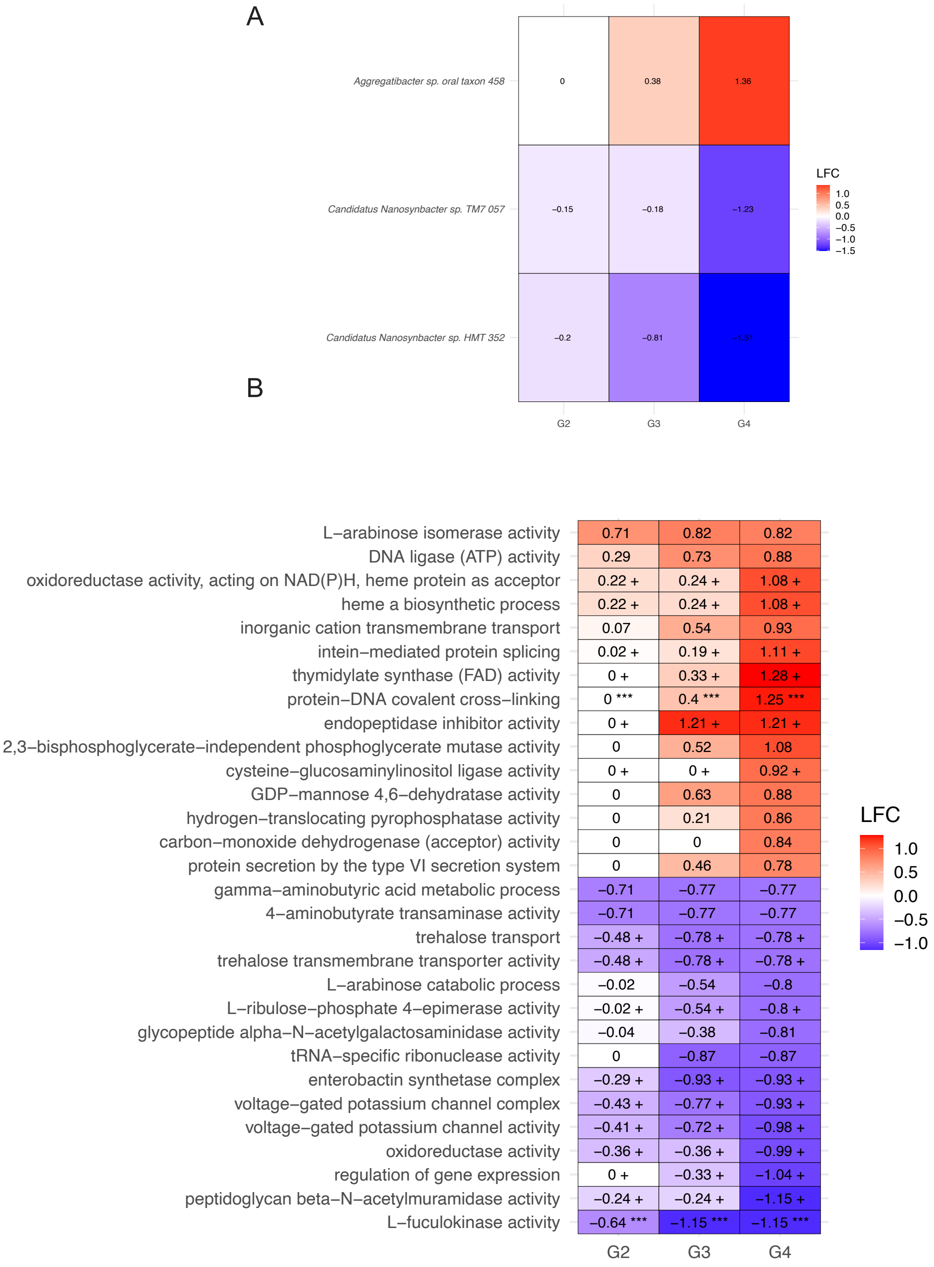

Supplementary Figure 5 (Trend analysis of oral microbiome over sexual activity groups). Trend analysis over four sexual activity groups defined as the number of partners with whom a participant had receptive anal intercourse: G_1, G_2, G_3, G_4 corresponds to groups with 0, 1, 2-5, and 6 or more receptive anal intercourse partners, respectively. Log-fold changes of absolute abundances of features in G_2, G_3, and G_4 relative to G_1 are presented for statistically significant differentially abundant features ($p < 0.01$) with + $q < 0.1$, * $q < 0.05$, ** $q < 0.01$, *** $q < 0.001$. The red and blue tiles indicate that the abundances of features have an increasing and decreasing trend over the sexual activity groups, respectively. These results are obtained by applying ANCOM-BC2. **A.** Oral bacterial species. **B.** Oral microbial Gene Ontology (GO) terms, only the top 30 significant GO terms are shown in this figure. (Pre-HIV n = 86, Non-HIV n = 151).

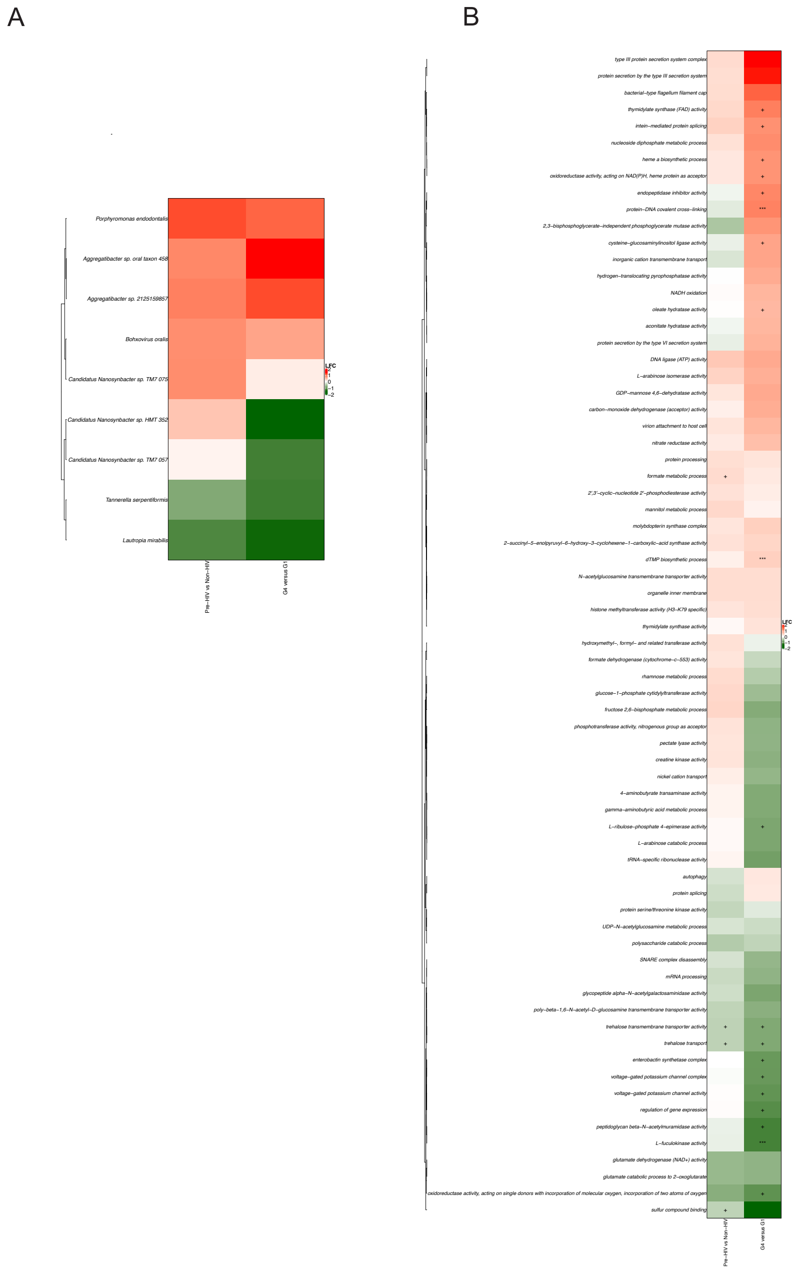

Supplementary Figure 6 (Differential abundance analysis of oral microbiome between Pre-HIV and Non-HIV versus trend analysis over sexual activity groups). Heatmaps show significant features at $p < 0.01$ from differential abundance analysis (DAA) between Pre-HIV and non-HIV and trend analysis over four sexual activity groups. + $q < 0.1$, * $q < 0.05$, ** $q < 0.01$, *** $q < 0.001$. For DAA, log-fold changes of absolute abundances of features in Pre-HIV relative to Non-HIV are presented. For trend analysis, although the p-values are derived using all four groups G_1, G_2, G_3, G_4, for graphical representation, the differences in the log-fold changes of absolute abundances in the extreme groups G_4 relative to G_1 (i.e., G_4 minus G_1) are presented. These results are obtained by applying ANCOM-BC2. **A.** Oral bacterial species. **B.** Oral microbial Gene Ontology (GO) terms.

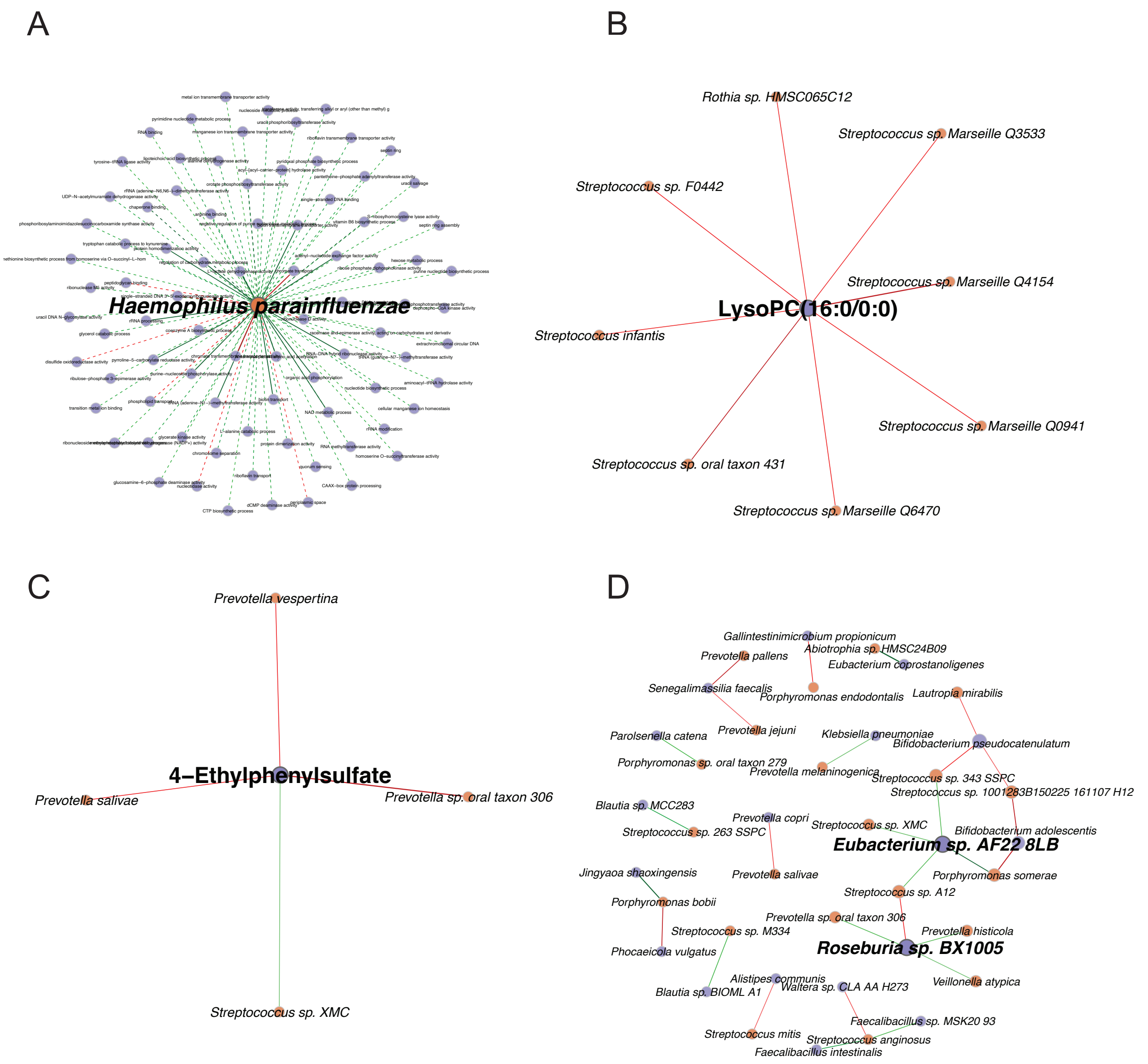

Supplementary Figure 7 (Differential correlation analysis related to oral microbiome between Pre-HIV and Non-HIV). Networks show a subset of significant differential Spearman correlations between Pre-HIV and Non-HIV with absolute difference in correlation > 0.3, $p < 0.01$, and $q < 0.1$ **A.** Differential Correlations between *Haemophilus parainfluenzae* and Gene Ontology (GO) terms. **B.** Differential correlations between 1-hexadecanoyl-sn-glycero-3-phosphocholine (LPC 16:0) and oral species. **C.** Differential correlations between 4-ethylphenylsulfate and oral species. **D.** Differential correlations between gut species within the genera *Eubacterium*, *Bifidobacterium*, and *Roseburia* and oral species within the genera *Streptococcus*, *Porphyromonas,* and *Prevotella*. Within each network, a pair of dots (nodes) is connected by an edge if the pair is differentially correlated between Pre-HIV and Non-HIV with $p < 0.01$, $q < 0.1$, the absolute difference in correlation more than 0.3. Correlations with $q < 0.05$ and $0.05 \leq q < 0.1$ are presented by solid lines and dashed lines, respectively. Green edges correspond to correlations that are higher in Pre-HIV compared to Non-HIV, and red edges correspond to correlations that are lower in Pre-HIV compared to Non-HIV. Brown nodes represent oral species, and purple nodes represent GO terms or metabolites. All Spearman correlation coefficients are derived after correcting for compositionality and adjusting for age, location, and antibiotic use as covariates. Correlation coefficients were corrected for different sample sizes of Pre-HIV and Non-HIV and were compared using two-sided Fisher's Z test. The sample sizes vary by data modality as described in Supplementary Data 12.

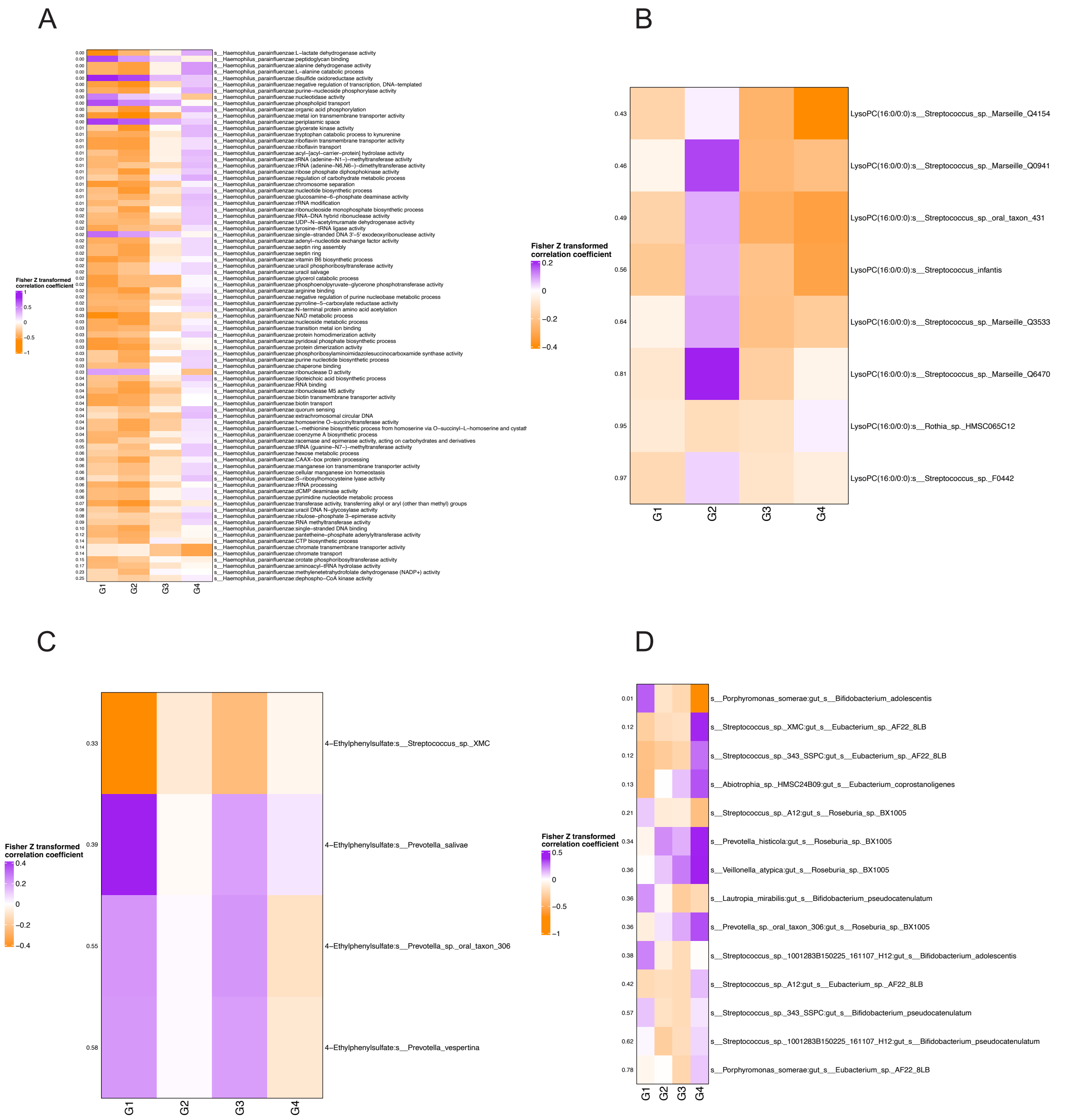

Supplementary Figure 8 (Heatmaps of correlations among features related to oral microbiome across sexual activity groups). Heatmaps show Spearman correlation coefficients of pairs of features that were differentially correlated between Pre-HIV and Non-HIV as shown in Supplementary Figure 7 for each sexual activity group. Benjamini-Hochberg adjusted p-values from the trend analysis (PAVA) are shown. **A.** Correlations between *Haemophilus parainfluenzae* and Gene Ontology (GO) terms. **B.** Correlations between 1-hexadecanoyl-sn-glycero-3-phosphocholine (LPC 16:0) and oral species. **C.** Correlations between 4-ethylphenylsulfate and oral species. **D.** Correlations between gut species within the genera *Eubacterium*, *Bifidobacterium*, and *Roseburia* and oral species with the genera *Streptococcus*, *Porphyromonas,* and *Prevotella*. The sample sizes vary by data modality as described in Supplementary Data 12.

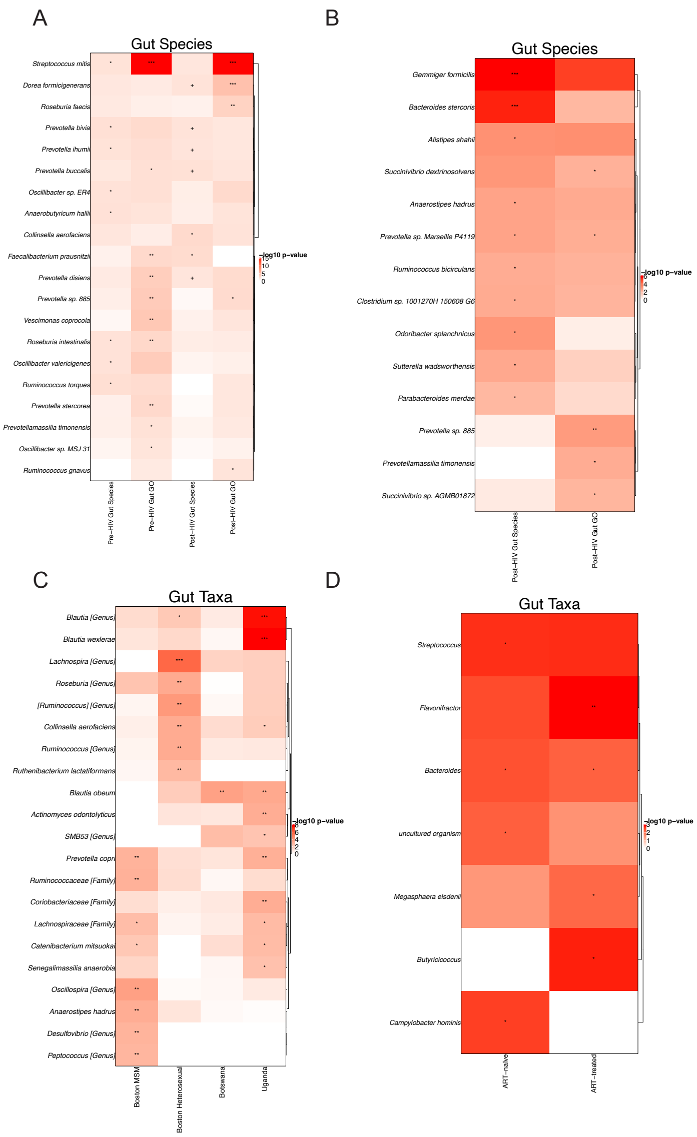

Supplementary Figure 9 (Application of DISCO to external datasets). Heatmaps show p-values from DISCO for four external datasets. In each heatmap, significantly disrupted species in each cohort or data modality are denoted by + $p < 0.01$ and $q < 0.1$, * $p < 0.01$ and $q < 0.05$, ** $p < 0.01$ and $q < 0.01$, *** $p < 0.01$ and $q < 0.001$. The number of correlations for each significant species is more than 20% of maximum correlations in a cohort or data modality. For each cohort or data modality the top 10 most significant species were selected for graphical representation. **A.** Fulcher et al. 2022: DISCO scores were computed for MSM Pre-HIV-1 and Post-HIV-1 (n = 27) versus matched controls without HIV-1 (n = 27) using gut species and Gene Ontologies (GO) characterized by metagenomic sequencing. **B.** Garcia et al. 2024: DISCO scores were computed for MSM with HIV-1 (n = 77) versus MSM without HIV-1 (n = 23) using gut species and Gene Ontologies (GO) characterized by metagenomic sequencing. **C.** Rocafort 2024 et al.: DISCO scores were computed for four different cohorts: MSM Boston cohort (n = 32 HIV-1 negative versus n = 86 HIV-1 positive), non-MSM Boston cohort (n = 85 HIV-1 negative versus n = 30 HIV-1 positive), Botswana cohort (n = 80 HIV-1 negative versus n = 114 HIV-1 positive), and Uganda cohort (n = 80 HIV-1 negative versus n = 90 HIV-1 positive). **D.** Armstrong et al. 2018: DISCO scores were computed for 32 MSM without HIV-1 versus MSM with HIV-1 who were ART- naïve (n = 39) and ART-treated HIV-1 positive (n = 50). Details on each study can be found in the Supplementary Methods.

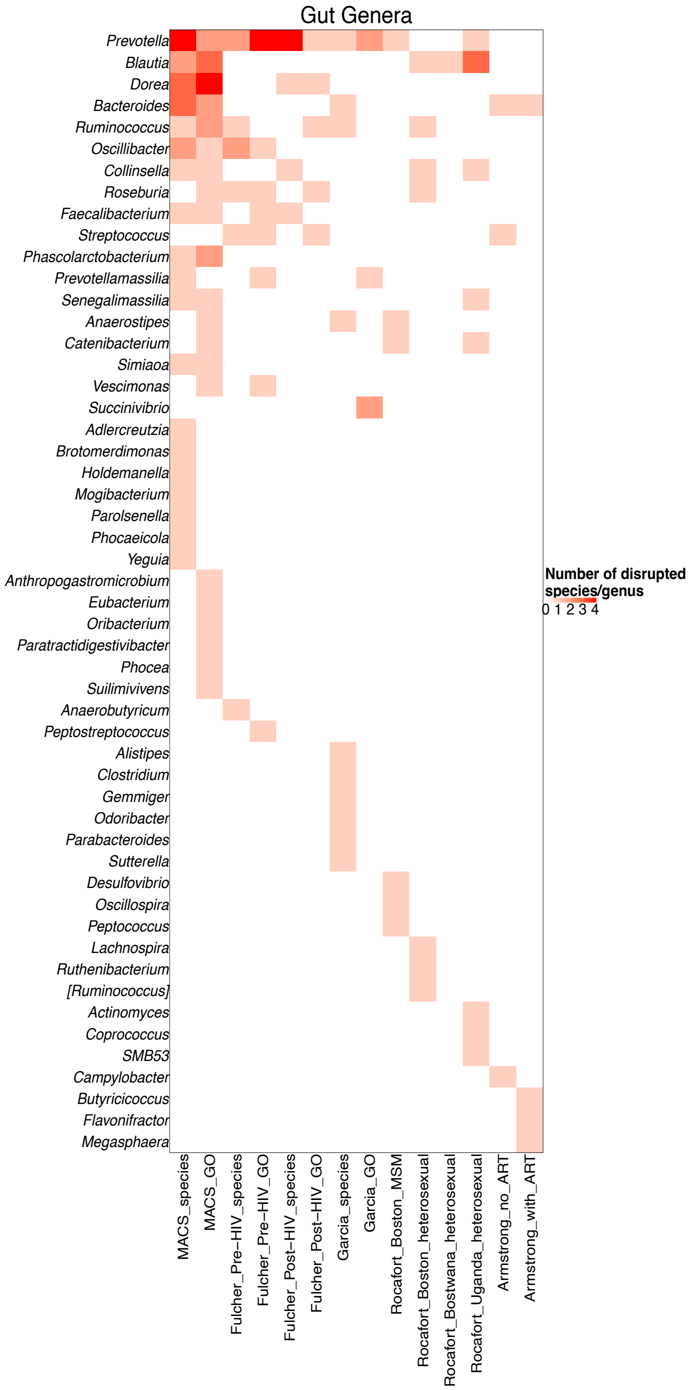

Supplementary Figure 10 (Heatmap of number of disrupted species across multiple cohorts). Heatmap show number of significant/suggestive disrupted species within each genus across different cohorts. Genera that are not classified to the species level are also included.

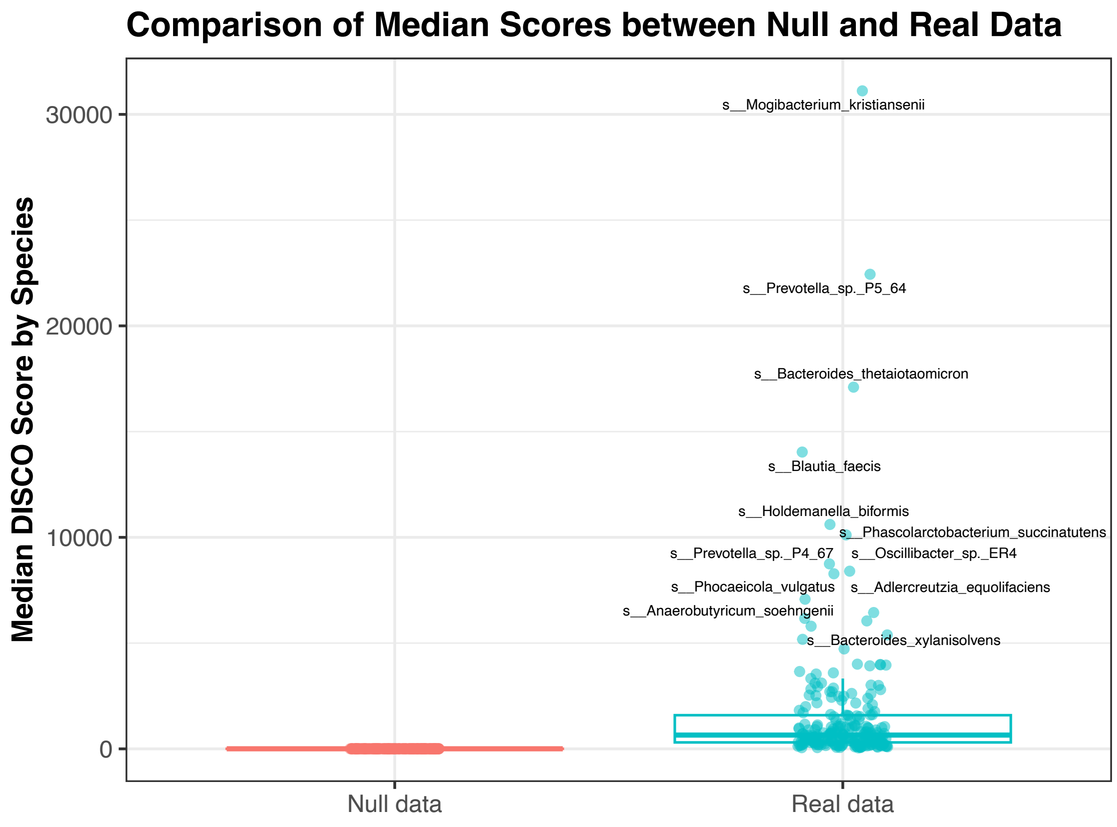

Supplementary Figure 11 (Validation study of DISCO). The boxplot shows the median DISCO scores over 1000 bootstrap samples with replacement from null or real data. To generate null data, the Pre-HIV group (n_1 = 86) and Non-HIV group (n_2 = 149) were combined into a single population and then were randomly resampled with replacement into two groups of sizes n_1 = 86 and n_2 = 149. For real data, a random sample with replacement of size 86 from the Pre-HIV group (n_1 = 86) and a random sample with replacement of size 149 from the Non-HIV group (n_2 = 149) were drawn in each iteration. The gut species abundance table from the MACS cohort was used for this analysis.

References

1 Kendlbacher, F. L. *et al.* Multispecies biofilm behavior and host interaction support the association of Tannerella serpentiformis with periodontal health. *Mol Oral Microbiol* **38**, 115-133 (2023). https://doi.org/10.1111/omi.12385

2 Gerner-Smidt, P. *et al.* Lautropia mirabilis gen. nov., sp. nov., a gram-negative motile coccus with unusual morphology isolated from the human mouth. *Microbiology (Reading)* **140 (Pt 7)**, 1787-1797 (1994). https://doi.org/10.1099/13500872-140-7-1787

3 van Winkelhoff, A. J., van Steenbergen, T. J. & de Graaff, J. Porphyromonas (Bacteroides) endodontalis: its role in endodontal infections. *J Endod* **18**, 431-434 (1992). https://doi.org/10.1016/s0099-2399(06)80843-5

4 Abusleme, L. *et al.* The subgingival microbiome in health and periodontitis and its relationship with community biomass and inflammation. *ISME J* **7**, 1016-1025 (2013). https://doi.org/10.1038/ismej.2012.174

5 Griffen, A. L. *et al.* Distinct and complex bacterial profiles in human periodontitis and health revealed by 16S pyrosequencing. *ISME J* **6**, 1176-1185 (2012). https://doi.org/10.1038/ismej.2011.191

6 Kikawada, T. *et al.* Trehalose transporter 1, a facilitated and high-capacity trehalose transporter, allows exogenous trehalose uptake into cells. *Proc Natl Acad Sci U S A* **104**, 11585-11590 (2007). https://doi.org/10.1073/pnas.0702538104

7 da Costa Morato Nery, D. *et al.* The role of trehalose and its transporter in protection against reactive oxygen species. *Biochim Biophys Acta* **1780**, 1408-1411 (2008). https://doi.org/10.1016/j.bbagen.2008.05.011

8 Ternes, D. *et al.* Author Correction: The gut microbial metabolite formate exacerbates colorectal cancer progression. *Nat Metab* **5**, 1638 (2023). https://doi.org/10.1038/s42255-023-00898-5

9 Hughes, E. R. *et al.* Microbial Respiration and Formate Oxidation as Metabolic Signatures of Inflammation-Associated Dysbiosis. *Cell Host Microbe* **21**, 208-219 (2017). https://doi.org/10.1016/j.chom.2017.01.005

10 Abranches, J. *et al.* Biology of Oral Streptococci. *Microbiol Spectr* **6** (2018). https://doi.org/10.1128/microbiolspec.GPP3-0042-2018

11 Ramanan, P., Barreto, J. N., Osmon, D. R. & Tosh, P. K. Rothia bacteremia: a 10-year experience at Mayo Clinic, Rochester, Minnesota. *J Clin Microbiol* **52**, 3184-3189 (2014). https://doi.org/10.1128/JCM.01270-14

12 Chattopadhyay, S. *et al.* Oral microbiome dysbiosis among cigarette smokers and smokeless tobacco users compared to non-users. *Sci Rep* **14**, 10394 (2024). https://doi.org/10.1038/s41598-024-60730-2

13 Halboub, E. *et al.* Tongue microbiome of smokeless tobacco users. *BMC Microbiol* **20**, 201 (2020). https://doi.org/10.1186/s12866-020-01883-8

14 Chipashvili, O. *et al.* Episymbiotic Saccharibacteria suppresses gingival inflammation and bone loss in mice through host bacterial modulation. *Cell Host Microbe* **29**, 1649-1662 e1647 (2021). https://doi.org/10.1016/j.chom.2021.09.009

15 Nie, J. *et al.* Strain-Level Variation and Diverse Host Bacterial Responses in Episymbiotic Saccharibacteria. *mSystems* **7**, e0148821 (2022). https://doi.org/10.1128/msystems.01488-21
16 Al-Kamel, A. *et al.* Subgingival microbiome of experimental gingivitis: shifts associated with the use of chlorhexidine and N-acetyl cysteine mouthwashes. *J Oral Microbiol* **11**, 1608141 (2019). https://doi.org/10.1080/20002297.2019.1608141
17 Huang, S. *et al.* Preliminary characterization of the oral microbiota of Chinese adults with and without gingivitis. *BMC Oral Health* **11**, 33 (2011). https://doi.org/10.1186/1472-6831-11-33
18 Hajishengallis, G. The inflammophilic character of the periodontitis-associated microbiota. *Mol Oral Microbiol* **29**, 248-257 (2014). https://doi.org/10.1111/omi.12065
19 Law, S. H. *et al.* An Updated Review of Lysophosphatidylcholine Metabolism in Human Diseases. *Int J Mol Sci* **20** (2019). https://doi.org/10.3390/ijms20051149
20 Needham, B. D. *et al.* Plasma and Fecal Metabolite Profiles in Autism Spectrum Disorder. *Biol Psychiatry* **89**, 451-462 (2021). https://doi.org/10.1016/j.biopsych.2020.09.025
21 Pascual, F., Camilli, S., Lockey, R. F. & Kolliputi, N. Mind-body connection: metabolite 4-ethylphenyl linked to anxiety behavior and oligodendrocyte modification in autism spectrum disorder. *Am J Physiol Gastrointest Liver Physiol* **324**, G422-G425 (2023). https://doi.org/10.1152/ajpgi.00221.2022
22 Armstrong, A. J. S. *et al.* An exploration of Prevotella-rich microbiomes in HIV and men who have sex with men. *Microbiome* **6**, 198 (2018). https://doi.org/10.1186/s40168-018-0580-7
23 Quast, C. *et al.* The SILVA ribosomal RNA gene database project: improved data processing and web-based tools. *Nucleic Acids Res* **41**, D590-596 (2013). https://doi.org/10.1093/nar/gks1219
24 Fulcher, J. A. *et al.* Gut dysbiosis and inflammatory blood markers precede HIV with limited changes after early seroconversion. *EBioMedicine* **84**, 104286 (2022). https://doi.org/10.1016/j.ebiom.2022.104286
25 Rubio Garcia, E. *et al.* Gut resistome linked to sexual preference and HIV infection. *BMC Microbiol* **24**, 201 (2024). https://doi.org/10.1186/s12866-024-03335-z
26 Rocafort, M. *et al.* HIV-associated gut microbial alterations are dependent on host and geographic context. *Nat Commun* **15**, 1055 (2024). https://doi.org/10.1038/s41467-023-44566-4
