# Supplementary material for: A taxon-specific measurement of disruption in a multi-modal study of microbiomes and metabolomes reveals system-wide dysbiosis preceding HIV-1 infection
Source: Nat Commun. 2025 Nov 20;16:10204. doi: 10.1038/s41467-025-64822-z (PMC12635353; doi:10.1038/s41467-025-64822-z)
Supplement: Supplementary file 2 — Description of Additional Supplementary Files [file 41467_2025_64822_MOESM2_ESM.pdf]

**Description of Additional Supplementary Files**

**File Name: Supplementary Data 1**
**Description: Differential abundance analysis (DAA) of gut microbiome and gut and plasma metabolites between Pre-HIV and Non-HIV. A-D.** Log-fold changes (LFC) of absolute abundances are presented for statistically significant differentially abundant features ($p < 0.01$). Positive LFC represents a higher abundance in Pre-HIV and negative LFC represents a lower abundance in Pre-HIV compared to Non-HIV. These results are obtained by applying ANCOM-BC2. $q$-values are adjusted $p$-values for multiple hypothesis testing using the Benjamini-Hochberg procedure. **A.** DAA of gut bacterial species. **B.** DAA of gut microbial Gene Ontology (GO) terms. **C.** DAA of gut metabolites. **D.** DAA of plasma metabolites. **E.** DAA of gut and plasma short chain fatty acids (SCFA). The significance of SCFA results is based on linear regression analysis. The sample sizes vary by data modality as described in Supplementary Data 12.

**File Name: Supplementary Data 2**
**Description: Trend analysis of gut microbiome and gut and plasma metabolites over sexual activity groups.** Trend analysis over four sexual activity groups defined as the number of partners with whom a participant had receptive anal intercourse: $G_1$, $G_2$, $G_3$, $G_4$ correspond to groups with 0, 1, 2-5, and 6 or more receptive anal intercourse partners, respectively. Log-fold changes of absolute abundances of features in $G_2$, $G_3$, and $G_4$ relative to $G_1$ are presented for statistically significant differentially abundant features ($p < 0.01$). These results are obtained by applying ANCOM-BC2. $q$-values are adjusted $p$-values for multiple hypothesis testing using the Benjamini-Hochberg procedure. **A.** Gut bacterial species. **B.** Gut microbial Gene Ontology (GO) terms. **C.** Gut metabolites. **D.** Plasma metabolites.

**File Name: Supplementary Data 3**
**Description: Multi-omics analysis**. Correlations between features obtained from Data Integration Analysis for Biomarker discovery using Latent cOmponents (DIABLO). Data modalities include gut species, gut microbial Gene Ontology (GO) terms (Gut_GO), gut metabolites from positive and negative ion channels (MTB_Gut), and plasma metabolites from positive and negative ion channels (MTB_Plasma). Features identified from the latent components 1 and 2 are included in this table. These results are obtained by applying DIABLO. For this analysis, samples that have all the three data modalities (gut microbiome, gut metabolites, and plasma metabolites) were included (Pre-HIV $n$ = 82, Non-HIV $n$ = 148).

**File Name: Supplementary Data 4**
**Description: Differential correlation analysis related to gut microbiome between Pre-HIV and Non-HIV.** Differential correlation analysis of pairs of features between Pre-HIV and Non-HIV are performed using Spearman Rank-Order correlation and two-sided Fisher's Z-transformation to test equality of correlations. $\rho_1$ and $\rho_2$ denote correlation coefficients in Non-HIV and Pre-HIV, respectively. $n_1$ and $n_2$ denote the number of co-occurrences of the pairs in Non-HIV and Pre-HIV, respectively. **A**. Inter gut species correlations. **B.** Correlations between gut species and gut Gene Ontology (GO) terms. **C.** Correlations between gut species and gut metabolites (negative ion channel). **D.** Correlations between gut species and gut metabolites (positive ion channel). **E.** Correlations between gut species and plasma metabolites (negative ion channel). **F.** Correlations between gut species and plasma metabolites (positive ion channel). The sample sizes vary by data modality as described in Supplementary Data 12.

**File Name: Supplementary Data 5**
**Description: Significant/Suggestive differential correlation analysis related to gut microbiome between Pre-HIV and Non-HIV.** Differential correlation analysis of pairs of features between Pre-HIV and Non-HIV are performed using Spearman Rank-Order correlation and two-sided Fisher's Z-transformation to test

equality of correlations. Only pairs of features with absolute difference in correlation (abs_diff_cor) > $0.3$, $p < 0.01$, and $q < 0.1$ are included in the table. For each pair, a trend analysis of correlation coefficients over the four sexual activity groups using the PAVA algorithm was performed. $\rho_1$ and $\rho_2$ denote correlation coefficients in Non-HIV and Pre-HIV, respectively. $n_1$ and $n_2$ denote the number of co-occurrences of the pairs in Non-HIV and Pre-HIV, respectively. $p$-value ($p$) was derived from the two-sided Fisher's Z-transformation to compare the correlation coefficients ($\rho_1$ and $\rho_2$). $\rho_{G_1}, \rho_{G_2}, \rho_{G_3}, \rho_{G_4}$ are Spearman correlation coefficients for each sexual activity group. E denotes the value of the trend test statistic. $p$-values from the trend test were adjusted using the Benjamini-Hochberg procedure. **A**. Inter gut species correlations. **B.** Correlations between gut species and gut Gene Ontology (GO) terms. **C.** Correlations between gut species and gut metabolites (negative ion channel). **D.** Correlations between gut species and gut metabolites (positive ion channel). **E.** Correlations between gut species and plasma metabolites (negative ion channel). **F.** Correlations between gut species and plasma metabolites (positive ion channel). The sample sizes vary by data modality as described in Supplementary Data 12.

**File Name: Supplementary Data 6**
**Description: Differential abundance analysis of oral microbiome and oral short chain fatty acids between Pre-HIV and Non-HIV**. **A-B.** Differential abundance analysis (DAA) between Pre-HIV and Non-HIV. A-B. Log-fold changes (LFC) of absolute abundances are presented for differentially abundant features ($p < 0.01$). Positive LFC represents a higher abundance in Pre-HIV and negative LFC represents a lower abundance in Pre-HIV compared to Non-HIV. $q$-values are adjusted $p$-values for multiple hypothesis testing using the Benjamini-Hochberg procedure. These results are obtained by applying ANCOM-BC2. **A.** DAA of oral bacterial species. **B.** DAA of oral microbial Gene Ontology (GO) terms. **C.** Oral short chain fatty acids (SCFA). The significance of results for oral (SCFA) is based on linear regression analysis. The sample sizes vary by data modality as described in Supplementary Data 12.

**File Name: Supplementary Data 7**
**Description: Trend analysis of oral microbiome and oral metabolites over sexual activity groups.** Trend analysis over four sexual activity groups defined as the number of partners with whom a participant had receptive anal intercourse: $G_1$, $G_2$, $G_3$, $G_4$ correspond to groups with 0, 1, 2-5, and 6 or more receptive anal intercourse partners, respectively. Log-fold changes of absolute abundances of features in $G_2$, $G_3$, and $G_4$ relative to $G_1$ are presented for differentially abundant features ($p < 0.01$). $q$-values are adjusted $p$-values for multiple hypothesis testing using the Benjamini-Hochberg procedure. These results are obtained by applying ANCOM-BC2. **A.** Oral bacterial species. **B.** Oral microbial Gene Ontology (GO) terms. **C.** Oral metabolites. The sample sizes vary by data modality as described in Supplementary Data 12.

**File Name: Supplementary Data 8**
**Description: Differential correlation analysis related to oral microbiome between Pre-HIV and Non-HIV.** Differential correlation analysis of pairs of features (including oral species) between Pre-HIV and Non-HIV are performed using Spearman Rank-Order correlation and two-sided Fisher's Z-transformation to test equality of correlations. $\rho_1$ and $\rho_2$ denote correlation coefficients in Non-HIV and Pre-HIV, respectively. $n_1$ and $n_2$ denote the number of co-occurrences of the pairs in Non-HIV and Pre-HIV, respectively. **A**. Inter oral species correlations. **B.** Correlations between oral species and oral Gene Ontology (GO) terms. **C.** Correlations between oral species and oral metabolites (negative ion channel). **D.** Correlations between oral species and oral metabolites (positive ion channel). **E.** Correlations between oral species and plasma metabolites (negative ion channel). **F.** Correlations between oral species and plasma metabolites (positive ion channel). **G.** Correlations between oral species and gut species. The sample sizes vary by data modality as described in Supplementary Data 12.

**File Name: Supplementary Data 9**
**Description: Significant/suggestive differential correlation analysis related to oral microbiome between Pre-HIV and Non-HIV.** Differential correlation analysis of pairs of features (including oral species) between Pre-HIV and Non-HIV are performed using Spearman Rank-Order correlation and two-sided Fisher's Z-transformation to test equality of correlations. Only pairs of features with absolute difference in correlation (abs_diff_cor) > 0.3, $p < 0.01$, and $q < 0.1$ are included in the table. For each pair, a trend analysis of correlation coefficients over the four sexual activity groups using the trend test. $\rho_1$ and $\rho_2$ denote correlation coefficients in Non-HIV and Pre-HIV, respectively. $n_1$ and $n_2$ denote the number of co-occurrences of the pairs in Non-HIV and Pre-HIV, respectively. $p$-value was derived from the two-sided Fisher's Z-transformation to compare the correlation coefficients ($\rho_1$ and$\rho_2$). $\rho_{G_1}$, $\rho_{G_2}$, $\rho_{G_3}$, $\rho_{G_4}$ are Spearman correlation coefficients for each sexual activity group. E denotes the value of the trend test statistic. $p$-values from the trend test were adjusted using the Benjamini-Hochberg procedure. **A.** Correlations between oral species and oral Gene Ontology (GO) terms. **B.** Correlations between oral species and oral metabolites (negative ion channel). **C.** Correlations between oral species and oral metabolites (positive ion channel). **D.** Correlations between oral species and plasma metabolites (negative ion channel). **E.** Correlations between oral species and plasma metabolites (positive ion channel). **F**. Correlations between oral species and gut species. The sample sizes vary by data modality as described in Supplementary Data 12.

**File Name: Supplementary Data 10**
**Description: DISCO scores.** DISCO scores, the corresponding $p$-values from standard Cauchy distribution, BH-adjusted $p$-values, and number of correlations for each species are included in this Table. DISCO scores are derived from: **A**. Inter gut species correlations. **B.** Correlations between gut species and gut Gene Ontology (GO) terms. **C.** Correlations between gut species and gut metabolites (negative ion channel). **D.** Correlations between gut species and gut metabolites (positive ion channel). **E.** Correlations between gut species and plasma metabolites (negative ion channel). **F.** Correlations between gut species and plasma metabolites (positive ion channel). **G**. Inter oral species correlations. **H.** Correlations between oral species and oral Gene Ontology (GO) terms. **I.** Correlations between oral species and oral metabolites (negative ion channel). **J.** Correlations between oral species and oral metabolites (positive ion channel). **K.** Correlations between oral species and plasma metabolites (negative ion channel). **L.** Correlations between oral species and plasma metabolites (positive ion channel). **M.** Correlations between oral species and gut species. The sample sizes vary by data modality as described in Supplementary Data
12.

**File Name: Supplementary Data 11**
**Description: Application of DISCO to external datasets.** DISCO scores, the corresponding $p$-values, BH-adjusted $p$-values, and number of correlations for each species are included in this table. **A-D**. Fulcher et al. 2022: DISCO scores were computed for MSM Pre-HIV-1 and Post-HIV-1 ($n$ = 27) versus matched controls without HIV-1 ($n$ = 27) using gut species and Gene Ontologies (GO) characterized by metagenomic sequencing. **E-F.** Garcia et al. 2024: DISCO scores were computed for MSM with HIV-1 ($n$ = 77) versus MSM without HIV-1 ($n$ = 23) using gut species and Gene Ontologies (GO) characterized by metagenomic sequencing. **G-J**. Rocafort 2024 et al.: DISCO scores were computed for four different cohorts: MSM Boston cohort ($n$ = 32 HIV-1 negative versus $n$ = 86 HIV-1 positive), non-MSM Boston cohort ($n$ = 85 HIV-1 negative versus $n$ = 30 HIV-1 positive), Botswana cohort ($n$ = 80 HIV-1 negative versus $n$ = 114 HIV-1 positive), and Uganda cohort ($n$ = 80 HIV-1 negative versus $n$ = 90 HIV-1 positive). **K-L**. Armstrong et al. 2018: DISCO scores were computed for 32 MSM without HIV-1 versus MSM with

HIV-1 who were ART- naïve ($n$ = 39) and ART-treated HIV-1 positive ($n$ = 50). Details on each study can be found in the Supplementary Methods.

**File Name: Supplementary Data 12.**
**Description:** Number of samples for each data modality.
