# Supplementary material for: A taxon-specific measurement of disruption in a multi-modal study of microbiomes and metabolomes reveals system-wide dysbiosis preceding HIV-1 infection
Source: Nat Commun. 2025 Nov 20;16:10204. doi: 10.1038/s41467-025-64822-z (PMC12635353; doi:10.1038/s41467-025-64822-z)
Supplement: Supplementary file 15 — Reporting Summary [file 41467_2025_64822_MOESM15_ESM.pdf]

nature portfolio

Corresponding author(s): Shyamal Peddada

Last updated by author(s): September 17, 2025

Reporting Summary

Nature Portfolio wishes to improve the reproducibility of the work that we publish. This form provides structure for consistency and transparency in reporting. For further information on Nature Portfolio policies, see our Editorial Policies and the Editorial Policy Checklist.

Statistics

For all statistical analyses, confirm that the following items are present in the figure legend, table legend, main text, or Methods section.

n/a	Confirmed	
☐	☒	The exact sample size (*n*) for each experimental group/condition, given as a discrete number and unit of measurement
☐	☒	A statement on whether measurements were taken from distinct samples or whether the same sample was measured repeatedly
☐	☒	The statistical test(s) used AND whether they are one- or two-sided<br>*Only common tests should be described solely by name; describe more complex techniques in the Methods section.*
☐	☒	A description of all covariates tested
☐	☒	A description of any assumptions or corrections, such as tests of normality and adjustment for multiple comparisons
☐	☒	A full description of the statistical parameters including central tendency (e.g. means) or other basic estimates (e.g. regression coefficient) AND variation (e.g. standard deviation) or associated estimates of uncertainty (e.g. confidence intervals)
☐	☒	For null hypothesis testing, the test statistic (e.g. *F*, *t*, *r*) with confidence intervals, effect sizes, degrees of freedom and *P* value noted<br>*Give P values as exact values whenever suitable.*
☒	☐	For Bayesian analysis, information on the choice of priors and Markov chain Monte Carlo settings
☒	☐	For hierarchical and complex designs, identification of the appropriate level for tests and full reporting of outcomes
☒	☐	Estimates of effect sizes (e.g. Cohen's *d*, Pearson's *r*), indicating how they were calculated

Our web collection on statistics for biologists contains articles on many of the points above.

Software and code

Policy information about availability of computer code

Data collection	Data were collected and processed using RStudio (2023.09.0+463) with R version 4.4.1 (2024-06-14), Platform: aarch64-apple-darwin20 , macOS 15.5
Data analysis	Data were analyzed using RStudio (2023.09.0+463) with R version 4.4.1 (2024-06-14), Platform: aarch64-apple-darwin20 , macOS 15.5

For manuscripts utilizing custom algorithms or software that are central to the research but not yet described in published literature, software must be made available to editors and reviewers. We strongly encourage code deposition in a community repository (e.g. GitHub). See the Nature Portfolio guidelines for submitting code & software for further information.

Data

Policy information about availability of data

All manuscripts must include a data availability statement. This statement should provide the following information, where applicable:

- Accession codes, unique identifiers, or web links for publicly available datasets
- A description of any restrictions on data availability
- For clinical datasets or third party data, please ensure that the statement adheres to our policy

Codes can be accessed from https://github.com/FarnazFouladi/Microbiome_Metabolomics_HIV.git. For raw data availability, please refer to the manuscript.

Research involving human participants, their data, or biological material

Policy information about studies with human participants or human data. See also policy information about sex, gender (identity/presentation), and sexual orientation and race, ethnicity and racism.

Reporting on sex and gender	All participants in this study were men.
Reporting on race, ethnicity, or other socially relevant groupings	All participants were Men who had sex with Men (MSM) from the Multi-Center AIDS Cohort Study (MACS)
Population characteristics	This study included 239 MSM enrolled in the MACS between 1984-1985 in four US cities: Pittsburgh (n = 43), Baltimore-Washington DC (n = 63), Chicago (n = 64), and Los Angeles (n = 69). All 239 participants were HIV-1-negative at their first, baseline MACS clinic visit as defined by lack of HIV-1 ELISA and Western blot antibodies, and HIV-1 RNA by rtPCR. Of these 239 participants, 87 were defined as being primary HIV-1 infected by testing positive for serum HIV-1 enzyme immunoassay and Western blot antibodies and confirmed positive for HIV-1 RNA by rtPCR, at their second MACS clinic visit approximately 6 months later. These 87 men were termed Pre-HIV-Infection (Pre-HIV), while the other 152 participants remained HIV-negative at their second MACS clinic visit (Non-HIV).
Recruitment	We did not recruit any subjects. We used samples from MACS.
Ethics oversight	The study was conducted with institutional review board approval from all participating institutions, all ethical regulations relevant to human research participants were followed, and informed consent was obtained.

Note that full information on the approval of the study protocol must also be provided in the manuscript.

Field-specific reporting

Please select the one below that is the best fit for your research. If you are not sure, read the appropriate sections before making your selection.

[x] Life sciences [ ] Behavioural & social sciences [ ] Ecological, evolutionary & environmental sciences

For a reference copy of the document with all sections, see nature.com/documents/nr-reporting-summary-flat.pdf

Life sciences study design

All studies must disclose on these points even when the disclosure is negative.

Sample size	NA. As noted above we are using existing samples.
Data exclusions	Individuals who had CCR5-delta32 homozygous genotype were not included in this study because they are naturally protected against HIV-1
Replication	NA
Randomization	NA
Blinding	NA

Reporting for specific materials, systems and methods

We require information from authors about some types of materials, experimental systems and methods used in many studies. Here, indicate whether each material, system or method listed is relevant to your study. If you are not sure if a list item applies to your research, read the appropriate section before selecting a response.

Materials & experimental systems

n/a	Involved in the study	
[x]	[ ]	Antibodies
[x]	[ ]	Eukaryotic cell lines
[x]	[ ]	Palaeontology and archaeology
[x]	[ ]	Animals and other organisms
[ ]	[x]	Clinical data
[x]	[ ]	Dual use research of concern
[x]	[ ]	Plants

Methods

n/a	Involved in the study	
[x]	[ ]	ChIP-seq
[x]	[ ]	Flow cytometry
[x]	[ ]	MRI-based neuroimaging

Clinical data

Policy information about clinical studies

All manuscripts should comply with the ICMJE guidelines for publication of clinical research and a completed CONSORT checklist must be included with all submissions.

Clinical trial registration	NCT00046280
Study protocol	Multicenter AIDS Cohort Study (MACS)
Data collection	This study included 239 Men who had Sex with Men (MSM) enrolled in the Multi-center AIDS Cohort Study (MACS) between 1984-1985 in four US cities: Pittsburgh (n = 43), Baltimore-Washington DC (n = 63), Chicago (n = 64), and Los Angeles (n = 69).
Outcomes	All 239 participants were HIV-1-negative at their first, baseline MACS clinic visit as defined by lack of HIV-1 ELISA and Western blot antibodies, and HIV-1 RNA by rtPCR. Of these 239 participants, 87 were defined as being primary HIV-1 infected by testing positive for serum HIV-1 enzyme immunoassay and Western blot antibodies and confirmed positive for HIV-1 RNA by rtPCR, at their second MACS clinic visit approximately 6 months later. These 87 men were termed Pre-HIV-Infection (Pre-HIV), while the other 152 participants remained HIV-negative at their second MACS clinic visit (Non-HIV).

Plants

Seed stocks	*Report on the source of all seed stocks or other plant material used. If applicable, state the seed stock centre and catalogue number. If plant specimens were collected from the field, describe the collection location, date and sampling procedures.*
Novel plant genotypes	*Describe the methods by which all novel plant genotypes were produced. This includes those generated by transgenic approaches, gene editing, chemical/radiation-based mutagenesis and hybridization. For transgenic lines, describe the transformation method, the number of independent lines analyzed and the generation upon which experiments were performed. For gene-edited lines, describe the editor used, the endogenous sequence targeted for editing, the targeting guide RNA sequence (if applicable) and how the editor was applied.*
Authentication	*Describe any authentication procedures for each seed stock used or novel genotype generated. Describe any experiments used to assess the effect of a mutation and, where applicable, how potential secondary effects (e.g. second site T-DNA insertions, mosiacism, off-target gene editing) were examined.*
